# Exploring the relationship between dietary patterns and obesity among Nigerian adults: a cross-sectional study

**DOI:** 10.1186/s12889-024-18792-4

**Published:** 2024-05-15

**Authors:** Asaolu Segun, Bo Zhang, Abiona Modupe Mary, Dennis Kibenja, Jie Ma, Seif Said, Idowu Adeniyi, Lamin F. Barrow

**Affiliations:** 1 Food Safety and Health Research Center, School of Public Health, Southern Medical University, Guangzhou, China; 2 Department Of Nursing and Public Health, Faculty of Health and Allied Science, Zanzibar University, Zanzibar, Tanzania; 3 Federal Ministry of Industry, Trade and Investment, Abuja, Nigeria; 4 The Department of Public and Environmental Health, School of Medicine and Allied Health Science, University of Gambia, Serekunda, The Gambia

**Keywords:** Adult, Dietary pattern, Nigeria, Obesity, Overweight, s

## Abstract

**Background/Objective:**

No previous study has investigated the association between dietary pattern and both general and abdominal obesity risk among adults in Nigeria. This study aimed to evaluate the associations between dietary patterns and the risk of obesity among adult age 18 and above in Ekiti State, Southwestern Nigeria.

**Methods and study designs:**

A total of 1003 adults were included in this cross-sectional study (males = 558; females = 445). Body mass index (BMI) and waist-to-hip (WHR) were used to assess general and abdominal obesity respectively and they were categorized using WHO recommendation. Partial correlation analyses were performed to assess the associations of dietary patterns with BMI and WHR. Prevalence ratio between dietary pattern and both general and abdominal obesity were calculated using Robust Poisson Regression.

**Results:**

The prevalence of general obesity among adults was 15.9%, (11.6% among men and 20.2% among women); abdominal obesity was 32.3% (28.9% among males and 44.5% among females). Four dietary patterns were identified; diversified traditional pattern; typical traditional pattern; milk and bread pattern and egg and fish pattern. Diversified traditional pattern was negatively associated with BMI (PR = 0.571, 95%CI: 0.360 ~ 0.905, *p* = 0.017), and typical traditional pattern was positively associated with BMI (PR = 1.561, 95% CI: 1.043 ~ 2.339, *p* = 0.031) and WHR in females (PR = 1.849, 95% CI: 1.256 ~ 2.721, *p* = 0.005). In comparison to those in the lowest quartile, adults with the highest quartile of the typical traditional pattern had a higher risk for abdominal obesity (PR = 1.849, 95%CI = 1.256 ~ 2.721, *p* = 0.020).

**Conclusion:**

This study reports an alarming increase in Obesity prevalence among Nigeria adults which is greatly influence by their lifestyle and eating pattern. We found out that a typical traditional food pattern was associated with a higher risk of both general and abdominal obesity, but a diverse traditional food pattern was associated to a reduced risk of general obesity.

## Introduction

The pervasive impact of obesity as a global epidemic manifest with staggering variations across world regions, nations, and specific population subsets within countries is a public health concern. Over the past two decades, a marked surge in overweight and obesity rates has been witnessed, notably in regions experiencing rapid economic development. In 2008, the escalation of these health concerns was evident, with more than 1.4 billion adults aged 20 years and older classified as overweight. Among them, an alarming statistic revealed over 200 million men and close to 300 million women grappling with obesity [1, 2]. This trend continued its alarming ascent, reaching unprecedented proportions in 2016, when the worldwide count of overweight adults aged 18 years and above surpassed 1.9 billion, with a staggering 650 million adults classified as obese [3] representing an average global adult populace of 13% (11% of men and 15% of women) [3]. However, unhealthy dietary habits, including the consumption of processed foods high in sugars, unhealthy fats, and refined carbohydrates are the major contributor of weight gain and obesity. These dietary patterns often lack essential nutrients while providing excessive calories, contributing to the development of obesity and related health conditions. In addition to diet, sedentary lifestyles characterized by low levels of physical activity also play a crucial role in the obesity epidemic and the rise of non-communicable diseases (NCDs). Limited regular exercise and physical inactivity does not only contributes to weight gain but also increases the risk of various chronic diseases such as diabetes, heart disease, and certain types of cancer [4]. This trend is emblematic of the burgeoning global health risks attributed to NCDs, particularly obesity, with developing nations bearing an increasingly burdensome share of this health crisis [5]. The trajectory of NCD prevalence exhibits a distressing upward surge, causing an estimated 36 million deaths annually and becoming the leading cause of morbidity and mortality worldwide [6].

However, the surge in high-fat, high-energy food consumption coupled with sedentary behaviors is intrinsically linked to the rapid pace of westernization, urbanization, and mechanization witnessed across numerous nations [4, 7]. This dietary shift has catalyzed a swift and concerning rise in obesity rates among both youth and adults, permeating even into low-income countries where it coexists paradoxically with chronic undernutrition [8]. Unhealthy lifestyles and poor eating habits are found among the leading causes for developing obesity and non-communicable diseases (NCD) such as type 2 diabetes, cardiovascular disease, and cancer [9]. An estimated 2.8 million individuals each year pass away from obesity-related problems throughout the world. Consequently, the estimates of the prevalence of obesity in West Africa range greatly throughout the continent and stand at 10.0% [10]. In sub-Sahara Africa (SSA), the prevalence of obesity varied from 3.5% in Eritrea to nearly 64% in Seychelles [11]. In Nigeria, a concerning trend emerges with prevalent unhealthy eating habits characterized by the consumption of fast foods, irregular meal patterns (especially skipping breakfast), excessive snacking, sugar-laden beverage consumption, and insufficient intake of fruits and vegetables [12, 13]. Alarmingly, the burden is much more affecting the wellbeing and livelihood of the population, which is because of her weak healthcare facility, high poverty rate, and population. Addressing this concerning health trajectory necessitates a fundamental shift towards improved dietary patterns and lifestyles, advocating for balanced nutrient-rich diets and healthy eating behaviors. Emphasizing the consumption of nutritionally dense foods in appropriate proportions stands as a crucial strategy to mitigate the progression of these debilitating medical conditions [14]. While some significant progress has been made in understanding the pattern of Nigerian diets, there are still some aspects that are not fully known or continue to be the subject of active investigation. The association between dietary patterns and obesity has not been explored in Nigeria, particularly through comprehensive dietary pattern analysis. Previous studies in the country have mainly focused on describing the frequency of consumption of different food groups or dietary habits, which may not provide a complete understanding of the relationship between diet and obesity. Additionally, there is a lack of research investigating the relationship between dietary patterns and obesity among adults aged 18 and above in Nigeria. To the best of our knowledge, this is the first study conducted in assessing the association between dietary pattern and analysis of general and abdominal obesity in Nigeria. By deriving distinct dietary patterns based on the overall dietary intake of participants, this approach allows for a more comprehensive assessment of how dietary habits contribute to obesity risk. Through this study, we seek to shed light on the complex interplay between diet and obesity in Nigeria's adult population. Hence, this study aimed to document current dietary patterns among Nigerian adults and to explore the association between dietary patterns and obesity.

## Materials and method

### Study design and population

This is a cross-sectional study that was conducted from July 2023 to September 2024. It was designed and carried out by focusing on adults aged 18 and above in Ekiti state, southwestern, Nigeria. Participation in the study was made voluntary, anonymous and confidential.

### Sample size

The sample size for this study was calculated using the formula n = [(Z_α_ + Z_β_)/C]^2^ + 3, where n is the sample size, Z_α_ is the standard normal deviate of α (0.05), Z_β_ is the normal standard deviate of β (0.20), r is the expected correlation coefficient(0.103). According to similar research conducted by Shu et al. [15], a correlation value of 0.103 was found in one of the identified dietary pattern and obesity. Based on the above, this value was used in our study for the calculation of the sample size.

α (0.05).

β (0.20).

r = 0.103.

*Z*_*α* =_ *1.9600*

*Z*_*β*_ = *0.8416*

*C* = *0.5* × *ln [(1* + *r)/ (1-r)]* = *0.1034*

*n* = *738. Which was adjusted by 10% contingency (74).*

*n* = *812, which was rounded up to 1000*

*Therefore n* = *1000 adult participants*

### Sampling technique

**Fig. 1:**
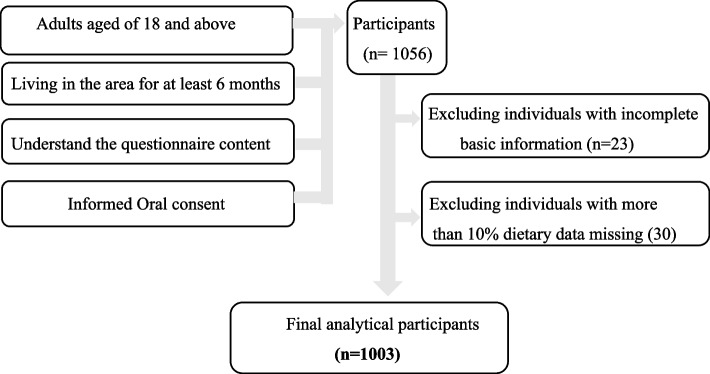
Flowchart of the selection of research participants

A multi-stage stratified cluster technique was used to select the study participants. (1) Eight local governments were randomly selected from the sixteen local governments. (2) A stratified sampling method was used to select two different towns from each local government. (3) Sixty-six eligible participants were randomly selected from each town. Eligible individuals were interviewed using an interview-administered questionnaire that was adopted from a related study [16]. The questionnaire comprised five sections: (i) Socioeconomic data (SWD); (ii) Lifestyle and physical activities pattern (LPA); (iii) Food habits (FH); (iv) Dietary pattern and frequency (DPF); and (v) Anthropometric data. The screening process for survey respondent is shown in Fig. 1.

### Data collection

All the instrument used in this survey were translated into the local language (Yoruba), which was further translated back into English language to ensure items retained their original meaning**.** Method of data collection comprised of personal interview and physical assessment (anthropometric measurements) of all participants. A well-structured and validated questionnaire was used to collect general information on food consumption pattern, dietary habits, lifestyle and physical activity, socio-economic and anthropometric data of subjects[17] The anthropometric measurement was assess by measuring height and weight to calculate body mass index (BMI), body circumference(waste and hip circumference) were also measured. Food Frequency Questionnaire was used for dietary assessment through examining the habitual food intake of subjects in the past one week. Furthermore, data were collected on the number of meals consumed daily, meal patterns, snacking habits, source of meals taken, alcohol intake, and food frequency consumption of ten food groups.

### Measurement of weight and height

Measurement of body weight was carried out using a pretested mechanical bathroom scale to the nearest 0.5 kg. This was done with shoes off and minimal clothing on. Scale was calibrated and recalibrated on daily basis by re-adjusting their pointers to zero. Height was measured in centimeters using a straight centimeter ruler with the patient standing erect on a flat surface (without shoes and heels) with buttocks and back of head touching the upright rod and height read to the nearest 0.1 m according to standard methods. [18]

### Determination of body mass index

Body mass index (BMI) was calculated as measured weight (kg) divided by measured height^2^ (m^2^). BMI is used as marker for nutritional status. BMI less than 18.5 kg/m2 is defined as underweight, normal weight is defined as BMI ranging from 18.5 to 24.9 kg/m^2^, BMI value of 25.0–29.9 kg/m^2^ defines overweight, while obesity is defined as BMI of 30.0 kg/m^2^ and above according to WHO classification[16].

### Determination of body circumferences

The waist and the hip circumferences were measured using a tape rule. To measure the waist circumference of each respondent, tape will be placed around bare abdomen just above the hip bone. Hip circumference was measured around the widest portion of the buttocks, with the tape parallel to the floor. It was ensured that the tape is snug, but not compressed or squeezed on skin and is parallel to the floor (Bruce, 2001). The waist to hip ratio was taken as the proportion of waist to hip measurements. According to WHO cut-off point, waist to hip value above 0.85 for women and 0.90 above for men will defined as obese [19].

### Dietary assessment

A Food Frequency Questionnaire (FFQ) was used to obtain the diet history of respondents using foods that are usually consumed by Southwest citizens of Nigeria. The FFQ contains 96 different foods consumed in the last 7 days preceding the study. The food frequency questionnaire included all the foods consumed daily by residents of southwestern part of Nigeria. It was adopted from related research conducted in Nigeria[16] and were collapsed into 17 food groups based on their nutritional profile as shown in Table 1. The questionnaire considered the frequency of food, drink, fruits and vegetables consumption, meal pattern, snacking habits and the frequency of consumption was represented by 6 categories namely, Never, 1 per day, 2–4 per day, 5–6 per week, 1 per day, 2–3 per day.

**Table 1:** Food grouping used in the factor analysis

Number	Food Groups	Example of food items
1	Swallow	Semo, Eba, Amala, Wheat, Fufu, Poundo yam
2	Legumes	Beans Porridge, Cowpea(Feregede), Bean Soup (Gbegiri), Beans Cake (Akara), Ekuru, Moinmoi
3	Starchy roots and tubers	Yam, yam porridge, cocoyam, sweet potatoes, cassava, Cassava flakes
4	Cereals and products	Spaghetti(Pasta), corn pap, Eko, Oats, Golden morn, corn flakes
5	Breads	White bread, coconut bread
6	Red meat	Beef, Pork, Chevon, Suya
7	White meat	Chicken, Turkey
8	Rice	White rice, jollof rice, fried rice, Ofada rice
9	Fruits	Pawpaw, Watermelon, Pineapple, Orange, Guava, Tangerine, Cucumber, Avocado Pear, Grapefruit, Mangoes, Banana, Coconut, African Cherry (Agbalumo), Apples
10	Vegetable	Carrot, Garden Egg, Cabbage, Vegetable Soup, Pepper And Tomato Stew, Okro, Corn, Ewedu, Egusi, Pumpkin, Mushroom
11	Oil and fats	Butter, Mayonnaise, Vegetable oil, Palm oil
12	Fish	All kinds of fish
13	Eggs	Eggs
14	Milk and related products	Cream Milk, Soya Milk, Yoghurts, Ice Cream, Powdered Milk
15	Beverages	Tea, Coffee, Bournvita/milo, fruit juice
16	Carbonated drinks	Coke, Fanta, malt etc.
17	Confectionaries	Cake, Puff Puff/buns/donut, Chinchin, Sharwama, Pizza, Burger, Chocolate Bar, Meat Pie, Fish Pie, Pop Corn,

### Physical activities assessment

Physical activity was measured using the Global Physical Activity Questionnaire (GPAQ-2) with 16 questions in it (P1-P16), and they evaluate sedentary behavior as well as three different types of physical activity: work, transportation, and leisure time physical activity [20]. Participants in this study were questioned whether they had engaged in vigorous, moderate or leisure time activities continuously for at least 10 min, the number of days engaging in each activity in a typical week, and the time spent in each activity in a typical day. The total time a person spends engaging in physical activity, or MET minutes per week, is determined from the answers to the frequency and duration questions. Activities with a vigorous intensity level cause people to breathe harder than normal, whereas activities with a moderate intensity level cause them to breathe slightly harder than normal. For adults, WHO global recommendation on physical activity for health is to do at least 150 min of moderate-intensity physical activity all over the week; or 75 min of vigorous-intensity physical activity all over the week; or an equivalent combination of moderate- and vigorous-intensity activity accumulating at least 600 MET-minutes per week [20]. In this study, we used this cut-off to define physically active (achieved 600 or more MET-minutes per week) versus inactive adults (achieving less than 600 MET-minutes per week) [21].

#### Dietary pattern establishment

To determine dietary patterns based on the frequency of consumption of all food classes in the FFQ, factor analysis (the primary component) was utilized. To create a more understandable structure, the factors were rotated using an orthogonal transformation (varimax rotation). The purpose of the factor analysis (or principal component analysis) is to identify underlying characteristics (or factors) that are responsible for the majority of the variance in the data by looking at the correlation matrix of dietary variables. As a result, a huge number of food factors are condensed into a smaller set of variables that accurately reflect the population's main dietary traits. The factors remained was determined using the eigenvalue (**> **1), scree plot, interpretability of the factors and their professional significance. Labeling of dietary patterns was based on the interpretation of foods with high factor loadings for each dietary pattern. Only foods with a factor loading ≥|0.3| was included in this study. The factor scores were further categorized into quartiles. The scores of comprehensive factors were divided into quartiles, with Q1, Q2, Q3, and Q4 representing the scores from lowest to highest, respectively.

#### Statistical analysis

The questionnaire was sorted and coded uniformly; data was presented as the mean ± standard deviation (SD) for continuous variables and as a sum (percentages) for categorical variables. Analysis of variance (ANOVA) to describe mean differences by continuous variables and the chi-squared test to evaluate the association between categorical variables. The characteristics of study participants was calculated across quartiles of each dietary pattern. Partial correlation analysis was performed to assess the associations between dietary patterns and BMI and WHR. Over 10% of people in this cross-sectional survey were obese. It could have overstated the relationship between obesity and independent variables if we had utilized the odd ratio. Hence, prevalence ratio is the most reliable indicator of connection in these situations and robust Poisson regression analysis was used to find obesity predictors. The 95% CI and prevalence ratio (PR) were computed, and the linear trends of the PRs were estimated. For every food pattern, one covariate-adjusted model and one unadjusted model were fitted. The first model is unmodified. Model 2 was adjusted for additional variables, such as age, monthly income, number of children, and educational attainment, based on the study and references [15, 22–24]. Two-sided ***p***-values < 0.05 were considered statistically significant. All analysis was performed on the Statistical Package for Social Sciences (version 23.0, SPSS Inc., Chicago, IL, USA).

## Result

### Participants characteristics

Overall, 1003 adults with complete data were included in this study (55.6% males, *n* = 558; 44.4% females, *n* = 445). As shown in Table 2, age range included is 18 years and above, in which they are categorized into 4 groups, 19-23 years (31.90%); 26-40 years (47.36); 41-65 years (16.15); 65 years and above (4.69%). In these age groups, the prevalence of obesity was highest among adults between the age 41–65 years. The overall prevalence of general obesity was 15.5% (11.6% in males; 20.2% in females) and abdominal obesity was 31.3% (29.6% in males and 33.0% in females), compared with non-obese adults, obese adults were more likely to be female, consumed alcohol, earned more than #50,000 monthly, married and self-employed (*p* **<** *0.05*).

**Table 2:** Demographic and lifestyle characteristics of the study participants

Variables	Total	BMI n (%)		***p***	Total	WHR (male) n (%)		***p***	Total	WHR (female) n (%)		***p***
		**non-obese**	**obese**			**non-obese**	**obese**			**non-obese**	**obese**	
**Sex**												
Male	558	493(88.4)	65(11.6)	**<0.001**	558	393(70.4)	165(29.6)	*a*	-	-	-	
Female	445	355(79.8)	90(20.2)						455	294(66.1)	151(33.9)	*a*
**Age**												
18-25	319	296(92.8)	23(7.2)	**<0.001**	169	106(62.7)	63(37.3)	**<0.001**	150	116(77.3)	34(22.7)	**0.001**
26-40	475	390(82.1)	85(17.9)		280	138(49.3)	142(50.7)		195	124(63.6)	71(36.4)	
41-65	162	123(75.9)	39(24.1)		83	30(36.1)	53(63.9)		79	43(54.4)	(45.6)	
65 and above	47	39(83.0)	8(17.0)		26	5(19.2)	21(63.9)		21	11(52.4)	10(47.6)	
**Religion**												
Christianity	834	711(85.3)	123(14.7)	0.437	457	228(49.9)	229(50.1)	0.273	377	253(67.1)	124(32.9)	0.267
Islam	153	123(80.4)	30(19.6)		87	47(54.0)	40(46.0)		66	39(59.1)	27(40.9)	
Traditional	14	12(85.7)	2(14.3)		12	4(33.3)	8(66.7)		2	2(100)	0(0.0)	
Others	2	2(100)	0(0.0)		2	-	2(100)		-	-	-	
**Marital status**												
Single	423	392(92.7)	31(7.33)	**<0.001**	261	152(58.2)	109(41.8)	**<0.005**	162	117(72.2)	45(27.8)	**0.039**
Married	556	436(78.4)	120(21.6)		287	122(42.5)	165(57.5)		269	171(63.6)	98(36.4)	
Divorced	2	2(100)	0(0.0)		1	2(100)	-		1	1(100)	-	
Widowed	13	9(69.2)	4(30.8)		5	3(60.0)	2(40.0)		8	2(25.0)	6(75)	
Others	9	9(100)	0(0.0)		4	9(50.0)	2(50.0)		5	3(60.0)	2(40)	
**How many kids,**												
1-3	426	330(77.5)	96(22.5)	**<0.001**	227	117(51.5)	110(48.5)	**<0.001**	199	127(63.8)	72(36.2)	0.144
4-6	146	118(90.8)	28(19.2)		72	14(19.4)	58(80.6)		74	43(58.1)	31(41.9)	
More than 6	12	12(100)	0(0.0)		6	3(50.0)	3(50.0)		6	4(66.7)	2(33.3)	
None	419	388(92.6)	31(7.4)		253	145(57.3)	108(42.7)		166	120(72.3)	46(27.7)	
**Occupation,**												
Employed	115	97(84.3)	18(15.7)	**<0.001**	78	23(29.5)	55(70.5)	**<0.001**	37	22(59.5)	15(40.5)	0.071
Self-employed	631	512(81.1)	119(18.9)		337	174(51.6)	163(48.4)		294	185(62.9)	109(37.1)	
Unemployed	11	9(81.8)	2(18.2)		7	3(42.9)	4(57.1)		4	3(75.0)	1(25.0)	
Student	231	218(94.4)	13(5.6)		125	77(61.6)	48(38.4)		106	82(77.4)	24(22.6)	
Retired	15	12(80.0)	3(20.0)		11	2(18.2)	9(81.8)		4	2(50)	2(50)	
**Level of education**												
Primary	80	64(80.0)	16(20.0)	0.412	41	15(36.6)	26(63.4)	0.192	39	23(59.0)	16(41)	0.589
Secondary	495	417(84.2)	78(15.8)		292	151(51.7)	141(48.3)		203	137(67.5)	66(32.5)	
Tertiary	428	367(85.7)	61(14.3)		225	113(50.2)	112(49.8)		203	134(66.0)	69(34.0)	
**Family type**												
Monogamous	798	682(85.5)	116(14.5)	0.113	433	213(49.2)	220(50.8)	0.477	365	248(67.9)	117(32.1)	0.074
Polygamous	205	166(81.0)	39(19.0)		125	66(52.8)	59(47.2)		80	46(57.5)	34(42.5)	
**Monthly income**												
Less than #20,000 ($50)	342	305(89.2)	37(10.8)	**0.006**	190	108(56.8)	82(43.2)	**<0.001**	152	111(73.0)	41(27.0)	0.083
#21,000-50,000 ($50-100)	578	479(82.9)	99(17.1)		314	158(50.3)	156(49.7)		264	165(62.5)	99(37.5)	
More than #50,000 ($100)	83	64(77.1)	19(22.9)		54	13(23.1)	41(75.9)		29	18(62.1)	11(37.9)	
**Alcohol**												
Yes	363	318(87.6)	45(12.4)	**0.044**	265	146(55.1)	119(44.9)	**0.022**	70	39(55.7)	31(44.3)	**0.046**
No	640	530(82.8)	110(17.2)		293	113(45.4)	160(54.6)		375	225(68.0)	120(32.0)	
**Sleeping hours per night**												
More than 7hrs.	740	613(82.8)	127(17.2)	**0.012**	385	193(50.1)	192(49.9)	0.927	355	239(67.3)	116(32.7)	0.266
Less than 7hrs.	263	235(89.4)	28(10.6)		173	86(49.7)	87(50.3)		90	55(61.1)	35(38.9)	
**Physical activity**												
Less than 600MET	133	111(83.5)	22(16.5)	0.709	59	24(40.7)	35(59.3)	0.176	74	53(71.6)	21(28.4)	0.269
600MET and above	870	737(84.7)	133(15.3)		499	255(51.1)	244(48.9)		371	241(65.0)	130(35.0)	

### Determination of dietary pattern

**Fig. 2:**
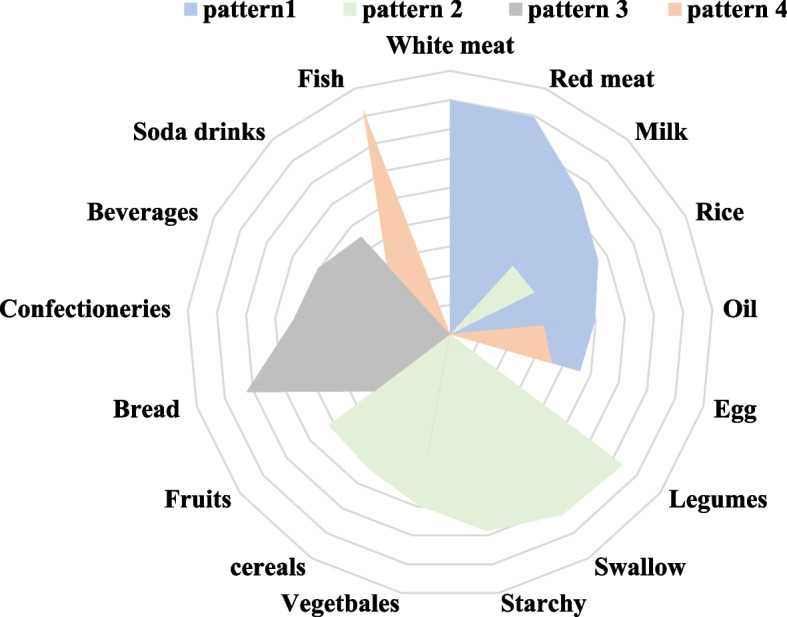
Rader chat of different dietary patterns obtained by factor analysis

Dietary pattern was determined using principal component analysis and the result are shown in Figure 2 and Table 3. Four different dietary patterns were generated and explained using factor loading and interpretability. Factor analysis identified four main dietary patterns from 19 food groups, which accounted for 31.09% (diversified food pattern), 10.51% (traditional pattern), 8.21% (milk and bread pattern), 5.89% (egg and fish pattern) of variance respectively, and together accounted for 55.7% of the total variance. After varimax rotation, the factor-loading matrix of the food groups was obtained and shown in Table 3. The diversified food pattern was characterized by meat, milk, rice and fruit. The traditional pattern was characterized by swallow, legumes, starchy food. The milk and bread pattern is characterized by milk, bread, and beverages. The egg and fish pattern is characterized by egg, fish and soda drinks.

**Table 3:** Factor loadings and dietary patterns for 17 food groups obtained by factor analysis

Food	Pattern I	Pattern II	Pattern III	Pattern IV
White meat	0.799			
Red meat	0.795			
Milk	0.655	0.317	0.360	
Rice	0.565	0.321		
Oil	0.497		0.362	0.320
Egg	0.462			0.362
Legumes		0.740		
Swallow		0.727		
Starchy		0.684		
Vegetables		0.595		0.416
cereals		0.536		
Fruits	0.470		0.324	
Bread			0.723	
Confectioneries		0.455	0.537	
Beverages	0.316		0.504	
Soda drinks			0.450	0.320
Fish				0.822

PCA (principal component analysis) was used for analysis. Factor analysis loading of < 0.3 in absolute terms were excluded for simplicity; pattern I: Diversified pattern; pattern II: traditional pattern; pattern III: milk and bread pattern; pattern IV: egg and fish pattern

### Characteristics of Quartiles (Q) of dietary patterns in study participants

The characteristics of the Q1 (lowest) and Q4 (highest) quartiles of the four dietary patterns are shown in Table 4. The analysis found that the percentage of males found in Q4 of pattern I, II, and IV is more than that of female except for pattern III. Adults in the highest quartiles of diversified pattern (pattern I) were more likely to be younger; had a greater portion of participants who are single; had higher educational levels; are from monogamous family; this participants do not have smoking, snacking and alcohol behavior; their sleeping hours is more than 7 h; they had lower BMI and WHR compared with participants in the lowest quartiles of pattern I (*p* < 0.001), while participants in the higher quartiles of traditional pattern (Pattern II) were more likely to be older, engaged in smoking alcohol behavior compare with pattern I. These participants were also more likely to be married; self-employed; earned averagely every month (#$50–100); usually skipped breakfast; had a greater portion of meal at dinner; sleeping hours is less than 7 h; they had higher BMI and WHR compared with participants in the lowest quartiles of pattern II (*p* < 0.001). Meanwhile, participants in the higher quartiles of milk and bread pattern (pattern III) were more likely to be widowed; have more than four children; had Tertiary education; self-employed and they are more likely from polygamous family compared to participants in the lowest quartiles of pattern III I (*p* < 0.001). Participants in the higher quartiles of egg and fish pattern (pattern IV) were more likely to be older, earned more than #50,000 ($100) monthly and they have longer sleeping duration compared with participants in the lowest quartiles of pattern IV (*p* < 0.00).

**Table 4:** Quartiles (Q) characteristics of dietary pattern scores in the study participants

	Pattern 1 n (%)		***p***	Pattern 2 n (%)		***p***	Pattern 3 n (%)		***p***	Pattern 4 n (%)		***p***
Variables	Q1	Q4		Q1	Q4		Q1	Q4		Q1	Q4	
**Gender**												
Male	129(43.1)	170(46.9)	**<0.001**	126(46.2)	147(53.8)	0.060	152(51.7)	142(48.3)	0.423	129(49.0)	134(51.0)	0.654
Females	121(60.2)	80(39.8)		125(54.6)	104(45.4)		99(48.1)	107(51.9)		121(51.1)	116(48.9)	
**Age**												
18-25yrs.	43(31.6)	93(68.4)	**<0.001**	99(63.3)	46(31.7)	**<0.001**	66(41.0)	95(59.0)	**<0.001**	114(67.5)	55(32.5)	**<0.001**
26-40yrs.	121(50)	121(50)		107(42.6)	144(57.4)		116(49.)	117(50.2)		106(47.1)	119(52.9)	
41-64yrs.	63(67.0)	31(33.0)		37(43.6)	49(57.4)		47(58.8)	33(41.3)		24(27.9)	62(72.1)	
≥65yrs	23(82.1)	5(17.9)		8(40.0)	12(60.0)		22(84.6)	4(15.4)		6(30.0)	14(70.0)	
**Religion**												
Christianity	213(50.7)	207(49.3)	0.807	210(50.5)	206(48.5)	0.611	210(51.0)	202(49.0)	0.123	215(51.1)	206(48.9)	0.155
Islam	34(47.2)	38(52.8)		38(48.1)	41(51.9)		33(42.3)	45(57.7)		29(40.8)	42(59.2)	
Traditional	2(33.3)	4(66.7)		2(33.3)	4(66.7)		7(77.8)	2(22.2)		4(66.7)	2(33.3)	
Others	1(50.0)	1(50.0)		1(100)	-		1(100.0)	-		2(100.0)	-	
**Marital status**												
single	61(32.1)	129(67.9)	**<0.001**	124(60.5)	81(39.5)	**0.001**	94(46.8)	107(53.2)	**0.003**	134(61.8)	83(38.2)	**<0.001**
married	180(61.2)	114(38.8)		120(42.0)	166(58.0)		51(65.4)	27(34.6)		112(41.5)	158(58.5)	
divorced	-	2(100)		-	1(100.0)		7(87.5)	1(12.5)		-	2(100.0)	
widowed	7(77.8)	2(22.2)		6(75.0)	2(25.0)		99(46.5)	114(53.5)		-	4(100.0)	
Others	2(40.0)	3(60.0)		1(50.0)	1(50.0)		251(50.2)	249(49.8)		4(57.1)	3(42.9)	
**How many kids**												
1-3	139(64.7)	76(35.3)	**<0.001**	101(44.5)	126(55.5)	**<0.001**	94(46.8)	107(53.2)	**0.003**	85(42.5)	115(57.5)	**<0.001**
4-6	51(56.7)	39(43.3)		26(37.1)	44(62.9)		51(65.4)	27(34.6)		28(34.6)	53(65.4)	
More than 6	-	5(100.0)		1(14.3)	6(85.7)		7(87.5)	1(12.5)		1(25.0)	3(75.0)	
none	60(31.6)	130(68.4)		123(62.1)	75(37.9)		99(46.5)	114(53.5)		136(63.3)	79(36.7)	
**Level of education**												
Primary	27(60.0)	18(40.0)	**<0.001**	13(28.3)	33(71.7)	**<0.001**	25(67.6)	12(32.4)	**0.004**	17(45.9)	20(54.1)	**<0.001**
secondary	158(64.8)	86(35.2)		126(45.5)	151(54.5)		138(54.1)	117(45.9)		84(35.9)	150(64.1)	
Tertiary	65(30.8)	146(69.2)		112(62.6)	67(37.4)		88(42.3)	120(57.7)		149(65.1)	80(34.9)	
**Occupation**												
Employed	30(46.9)	34(53.1)	**<0.001**	27(49.1)	28(50.9)	**<0.001**	34(60.7)	22(39.3)	**0.008**	18(32.1)	38(67.9)	**<0.001**
Self-employed	180(57.7)	132(42.3)		134(41.4)	190(58.6)		151(46.9)	171(53.1)		151(47.2)	169(52.8)	
Unemployed	1(33)	4(4)		1(16.7)	5(83.3)		1(50.0)	1(50.0)		1(50.0)	1(50.0)	
Student	33(30.0)	77(70.0)		83(75.5)	27(24.5)		55(50.0)	55(50.0)		77(66.4)	39(33.6)	
**Family type**												
Monogamous	180(44.9)	221(55.1)	**<0.001**	210(55.1)	171(44.9)	**<0.001**	200(50.5)	196(49.5)	0.790	215(51.7)	201(48.3)	0.94
Polygamous	70(70.7)	29(29.3)		41(33.9)	80(66.1)		51(49.0)	53(51.0)		35(41.7)	49(58.3)	
**Monthly income**												
Less than #20,000 ($50)	80(52.6)	72(47.4)	0.385	107(59.1)	74(40.9)	**<0.002**	88(52.4)	80(47.6)	0.429	96(56.5)	74(43.5)	**0.003**
#21,000-50,000 ($50-100)	157(49.8)	158(50.2)		121(42.9)	161(57.1)		138(47.9)	150(52.1)		140(49.8)	141(50.2)	
More than #50,000 ($100)	13(39.4)	20(60.6)		23(59.0)	16(41.0)		25(56.8)	19(43.2)		14(28.8)	35(71.4)	
**Alcohol**												
Yes	162(51.8)	151(48.2)	<0.309	175(54.9)	144(45.1)	**0.004**	146(46.6)	167(53.4)	**0.040**	174(54.4)	146(45.6)	**0.009**
No	88(47.1)	99(52.9)		76(41.5)	107(58.5)		105(56.1)	82(43.9)		76(43.3)	104(57.8)	
**Sleeping hours per night**												
<7hrs.	162(51.8)	151(48.2)	**<0.001**	169(45.6)	202(54.4)	**<0.001**	166(45.1)	202(54.9)	**<0.001**	186(51.2)	177(48.8)	0.367
>7hrs.	88(47.1)	99(52.9)		82(62.6)	49(37.4)		85(64.4)	47(35.6)		64(46.7)	73(53.3)	
**Physical activity**												
Less than 600MET	213(57.4)	158(42.6)	0.057	51(78.5)	14(21.5)	**<0.001**	44(62.9)	26(37.1)	**0.022**	33(44.0)	42(56.0)	0.260
600MET and above	37(28.7)	92(71.3)		200(45.8)	237(54.2)		207(48.1)	233(51.9)		217(51.1)	208(48.9)	
**BMI**												
Non-obese	205(82.0)	226(90.4)	**0.031**	217(86.5)	34(13.5)	**0.006**	224(89.6)	26(10.4)	0.66	218(86.9)	33(13.1)	0.704
Obese	45(18.0)	24(9.6)		195(77.7)	56(22.3)		211(84.4)	39(15.6)		209(83.9)	40(16.1)	
**WHR(male)**												
Non-obese	187(74.8)	63(25.2)	0.741	207(82.5)	44(17.5)	**0.004**	186(74.1)	65(25.9)	0.910	198(79.2)	52(20.8)	0.200
Obese	193(77.2)	57(22.8)		173(68.9)	78(31.1)		190(76.3)	59(23.7)		186(74.4)	64(25.6)	
**WHR(female)**												
**Non-obese**	145(58.0)	104(42.0)	0.489	171(68.1)	80(31.9)	**<0.001**	155(62.0)	95(38.0)	0.230	138(55.0)	113(45.0)	0.190
**Obese**	135(54.0)	115(46.0)		127(50.6)	124(49.4)		139(55.6)	111(44.4)		153(61.4)	96(38.6)	

### Relationship between dietary pattern score, BMI, and WHR using partial correlation analysis

Relationship between the four identified dietary patterns and obesity indicators (BMI and WHR) are shown in Table 5. After adjusting age, monthly income, how many kids and educational level, the Pearson correlation result indicated that diversified pattern had a negative correlation with BMI (*r* = -0.076, *p* =  < 0.05) while traditional dietary pattern had a positive correlation with BMI (*r* = 0.087, *p* =  < 0.05). Traditional pattern also had a positive correlation with WHR in both male and female (*r* = 0.096, r = 0.102, respectively, *p* =  < 0.05). Although the coefficients of correlation between pattern I and II were statistically significant, the association between dietary pattern score and the indicators of obesity was weak.

**Table 5:** Partial correlation analysis for the relationship between dietary pattern score, BMI, and WHR

Dietary pattern Score	BMI(Kg/m^**2**^)	***p***	WHR (Male)	***p***	WHR (female)	***P***
Pattern 1	-0.076	**0.016**	-0.10	0.747	0.037	0.247
Pattern 2	0.087	**0.006**	0.096	**0.002**	0.102	**0.001**
Pattern 3	0.029	0.360	-0.019	0.546	-0.059	0.064
Pattern 4	0.048	0.127	0.066	0.052	0.072	0.220

*Abbreviation:* *BMI *Body mass index, *WHR *Waist to hip ratio. Adjusted for age, monthly income, how many kids and educational level. Pattern I: Diversified pattern; pattern II: traditional pattern; pattern III: milk and bread pattern; pattern IV: egg and fish

### Analysis of association between dietary patterns and general obesity

The result of the relationship between dietary patterns and obesity (BMI) using Robust Poisson regression analysis is displayed in Table 6. The Mantel–Haenszel chi-square test-results show that there was a linear relationship between the tendency level of the diversified pattern, traditional pattern and BMI ( *p* =  < 0.05). Robust Poisson regression analysis showed that the high-tendency group (Q4) of diversified pattern (Pattern I) had a lower chance of becoming obese compared to the participants in the low-tendency group (Q1) for unadjusted model (PR = 0.571, 95% CI: 0.360 ~ 0.905, *p* = 0.017); but it showed no association after adjusting for the possible confounders in model 2; compared with the low-tendency group (Q1) of traditional pattern (Pattern II), the high tendency group (Q4) had a higher chance of becoming obese in both adjusted and unadjusted model (PR = 1.561, 95%CI: 1.043 ~ 2.339 in Model 1; PR = 1.506, 95%CI: 1.016 ~ 2.233 in Model 2 respectively, ( *p* =  < 0.05). Nevertheless, pattern II and III show no association with t Obesity.

**Table 6:** Analysis of the association between dietary pattern and BMI

Dietary pattern	Non-obese	obese	***p***	Model 1	***p***	Model 2	***p***
				PR (95%CI)		PR (95%CI)	
**Pattern 1, n (%)**							
**Q1**	205(82.0)	45(18.0)	**0.031**	1		1	
**Q2**	209(83.3)	42(16.7)		0.989(0.675, 1.448)	0.954	1.166(0.795, 1.711)	0.431
**Q3**	208(82.5)	44(17.5)		1.048(0.722, 1.524)	0.801	1.276(0.875, 1.861)	0.205
**Q4**	226(90.4)	24(9.6)		0.571(0.360, 0.905)	**0.017**	0.754(0.474, 1.199)	0.232
**Pattern 2, n (%)**							
**Q1**	217(86.5)	13.5(13.5)	**0.006**	1		1	
**Q2**	220(88.0)	30(12.0)		0.938(0.592, 1.488)	0.787	0.930(594, 1.456)	0.751
**Q3**	216(86.1)	35(13.9)		1.093(0.705, 1.693)	0.692	1.062(0.690, 1.633)	0.784
**Q4**	195(77.7)	56(22.3)		1.561(1.043, 2.339)	**0.031**	1.506(1.016, 2.233)	**0.041**
**Pattern 3, n (%)**							
**Q1**	218(86.9)	33(13.1)	0.704	1		1	
**Q2**	210(84.0)	40(16.0)		1.206(0.781, 1.862)	0.781	1.663(1.068, 2.593)	0.322
**Q3**	211(83.4)	42(16.6)		1.170(0.755, 1.814)	0.495	1.549(0.992, 2.417)	0.422
**Q4**	209(83.9)	40(16.1)		1.211(0.792, 1.850)	0.713	1.312(0.854, 2.015)	0.215
**Pattern 4, n (%)**							
**Q1**	224(89.6)	26(10.4)	0.66	1		1	
**Q2**	206(82.1)	45(17.9)		1.476(0.928, 2.348)	0.170	1.663(1.068, 2.592)	0.213
**Q3**	207(82.1)	45(17.9)		1.620(1.038, 2.527)	0.340	1.549(0.992, 2.417)	0.061
**Q4**	211(84.5)	155(15.5)		1.741(1.101, 2.689)	0.100	1.273(0.796, 2.034)	0.313

The chi-square trend test and robust Poisson regression analysis were used for analysis. Q1 was the reference group

Model 1 is unadjusted; Model 2: adjusted for age, monthly income, how many kids and educational level. The bold p-value means “ < 0.05”

### Analysis of association between dietary patterns and abdominal obesity

The result of the relationship between dietary patterns and abdominal obesity for both male and female using Robust Poisson regression analysis are displayed in Tables 7 and 8 respectively. The Mantel–Haenszel chi-square test-results show that there was no linear relationship between the derived dietary patterns and WHR in male; compared to male participants, there was a linear relationship between female participants and traditional pattern ( *p* =  < 0.05). As shown in Table 8, Robust Poisson regression analysis showed that the high-tendency group (Q4) of Traditional pattern (Pattern II) had a higher chance of becoming obese compared to the participants in the low-tendency group (Q1) for unadjusted model and adjusted model (PR = 1.849, 95% CI: 1.256 ~ 2.721 respectively, ( *p* =  < 0.05).

**Table 7:** Analysis of the association between dietary pattern and WHR in male

Dietary pattern	Total n (%)		***p***	Model 1 n (%)	***p***	Model 1 n (%)	
	Non-obese	Obese		PR (95%CI)		PR (95%CI)	
**Pattern 1**							
**Q1**	85(65.9)	44(34.1)	0.305	1		1	
**Q2**	93(75.0)	31(25.0)		0.712(0.481, 1.053)	0.182	0.803(0.549, 1.174)	0.257
**Q3**	91(67.4)	44(32.6)		0.945(0.665, 1.343)	0.753	1.092(0.776, 1.536)	0.615
**Q4**	124(72.9)	46(27.1)		0.787(0.553, 1.119)	0.182	0.955(0.680, 1.340)	0.789
**Pattern 2**							
**Q1**	95(75.4)	31(24.6)	0.254	1			
**Q2**	107(70.4)	45(29.6)		1.153(0.778, 1.709)	0.118	1.225(0.843, 1.781)	0.853
**Q3**	96(72.2)	37(27.8)		1.089(0.724, 1.637)	0.683	1.026(0.689, 1.526)	0.901
**Q4**	95(64.6)	52(35.4)		1.358(0.925, 1.993)	0.447	1.225(0.842, 1.781)	0.289
**Pattern 3**							
**Q1**	103(67.8)	49(32.2)	0.746	1		1	
**Q2**	105(73.4)	38(26.6)		0.888(0.626, 1.259)	0.503	0.947(0.669, 1.341)	0.413
**Q3**	84(69.4)	37(30.6)		0.902(0.628, 1.295)	0.576	0.010(0.713, 1.432)	0.766
**Q4**	101(71.1)	41(28.9)		0.847(0.595, 1.207)	0.359	1.029(0.731, 1.449)	0.466
**Pattern 4**							
**Q1**	96(74.4)	33(25.6)	0.105	1		1	
**Q2**	105(75.5)	34(25.4)		0.886(0.580, 1.355)	0.578	0.855(0.562, 1.302)	0.466
**Q3**	107(68.6)	49(31.4)		1.142(0.779, 1.674)	0.498	1.057(0.732, 1.527)	0.766
**Q4**	85(63.4)	49(36.6)		1.376(0.949, 1.993)	0.092	1.164(0.809, 1.675)	0.413

The chi-square trend test and robust Poisson regression analysis were used for analysis. Q1 was the reference group

Model 1 is unadjusted; Model 2: adjusted for age, monthly income, how many kids and educational level. The bold p-value means “ < 0.05”

**Table 8:** Analysis of the association between dietary pattern and WHR in female

Dietary pattern	Total n (%)		***p***	Model 1 n (%)	***p***	Model 2 n (%)	***p***
	Non-obese	Obese		PR (95%CI)		PR (95%CI)	
**Pattern 1**							
**Q1**	82(67.8)	39(32.2)	0.853	1		1	
**Q2**	80(63.0)	47(37.0)		1.349(0.959, 1.897)	0.777	1.574(1.103, 2.246)	0.210
**Q3**	78(66.7)	39(33.3)		1.100(0.771, 1.570)	0.598	1.289(0.899, 1.849)	0.167
**Q4**	54(67.5)	26(32.5)		1.062(0.699, 1.614)	0.085	1.248(0.805, 1.934)	0.344
**Pattern 2**							
**Q1**	96(76.8)	29(23.2)	**0.007**	1		1	
**Q2**	61(62.2)	37(37.8)		1.672(1.117, 2.502)	0.103	1.747(1.187, 2.571)	0.210
**Q3**	79(66.9)	39(33.1)		1.429(0.952, 2.143)	0.305	1.383(0.929, 2.060)	0.110
**Q4**	58(55.8)	46(44.2)		1.849(1.256, 2.721)	**0.020**	1.658(1.118, 2.460)	**0.005**
**Pattern 3**							
**Q1**	67(67.7)	32(32.3)	0.231	1		1	
**Q2**	62(57.9)	45(42.1)		1.251(0.878, 1.783)	0.819	1.263(0.889, 1.795)	0.192
**Q3**	90(68.2)	42(31.8)		0.912(0.623, 1.335)	0.634	0.950(0.648, 1.393)	0.794
**Q4**	75(70.1)	32(29.9)		0.955(0.45, 1.415)	0.819	1.027(0.693, 1.521)	0.896
**Pattern 4**							
**Q1**	88(72.7)	33(27.3)	0.303	1		1	
**Q2**	74(66.1)	38(33.9)		1.162(0.789, 1.711)	0.967	1.094(0.746, 1.504)	0.646
**Q3**	49(51.0)	47(49.0)		1.720(1.204, 2.458)	0.300	1.566(1.094, 2.243)	0.014
**Q4**	83(71.6)	33(28.4)		1.009(0.675, 1.507)	0.967	0.886(0.588, 1.337)	0.565

The chi-square trend test and robust Poisson regression analysis were used for analysis. Q1 was the reference group

Model 1 is unadjusted

## Discussion

Obesity stands as a formidable public health challenge, posing a threat to the well-being of billions across the globe [25]. Its pervasive nature renders it a critical risk factor for a spectrum of chronic illnesses, including cardiovascular diseases, type 2 diabetes, hypertension, and various cancers [26]. This study was conducted to assess the relationship between dietary patten and obesity among Nigerian adults. In 2014, a study conducted by Adebayo and colleagues in Oyo state (southwestern part of Nigeria) found that the prevalence of general obesity among adult aged 18 and above was 8.4% [27]. Another study conducted in 2018 found that the prevalence of general obesity among adults in Abia state (southeastern Nigeria) was 12.4% [28]. In this survey, the overall prevalence of general obesity among participants was 15.9%, with a prevalence of 11.6% in men and 20.2% in women; prevalence of abdominal obesity was 31.3%, with a prevalence of 29.6% among men and 33.9% among women. The observed differences between our study and previous studies mentioned above might be due to the reported rising level of overweight and obesity among Nigerian adults that requires an urgent attention. Some key reasons for this trend are rapid urbanization and adoption of western lifestyles which have led to changes in dietary habits and physical activity levels. Urbanization also comes with a shift towards sedentary occupations and lifestyles. Many people in urban areas spend long hours sitting at desks or commuting in cars, leading to decreased physical activity levels. Reduced physical activity, combined with unhealthy dietary habits, contributes to weight gain and obesity. More so, the availability and affordability of processed foods and sugary drinks have increased in Nigeria, contributing to higher calorie intake and weight gain. In this cross-sectional study, we identified four dietary patterns; including a diversified traditional pattern (pattern I), a typical traditional pattern (pattern II), a milk and bread pattern (pattern III), and an egg and fish pattern (pattern IV). These identified dietary patterns were similar to those reported previously among adults in other studies [29–33]. Pattern I and II had strong positive correlations with foods that are traditional in southwest Nigeria, hence they are named as “traditional”. In our study, the characteristics of Diversified traditional dietary pattern was high intake of fruits, milk, rice, white meat, red meat, beverages and oil. In this present study, we found an inverse relationship between this pattern and general obesity. Its cumulative contribution rate was the highest (31.09%). This pattern is similar to those reported in previous studies conducted among adults in Nigeria [34, 35], and also similar to those derived from other countries [36–38]. The protective effect observed in this dietary pattern can be attributed to its inclusion of healthy constituents such as rice, milk, oil, eggs, beverages, and fruits. Rice, fresh vegetables, and fruits are rich sources of dietary fiber, which has been consistently associated with a reduced risk of obesity in previous studies [39]. Certain foods in this traditional diversified pattern, such as milk and egg, have a low glycemic index (GI), which is linked to a decreased risk of obesity.

Furthermore, high consumption of beverages (tea, coffee, milo), milk and related product was observed in this pattern. Dairy products have long been a subject of interest in discussions surrounding adult obesity, given their diverse nutrient profile and potential impact on weight regulation. Research findings on the association between dairy consumption and obesity have been varied, with some studies suggesting a protective effect of dairy intake against obesity, while others indicate no significant association or even a potential risk. A systematic review and meta-analysis conducted by Lu and colleagues examined the long-term association between dairy consumption and the risk of obesity [40]. The study found that higher intake of dairy products, particularly low-fat dairy, was associated with a reduced risk of obesity and overweight. The authors speculated that the protein, calcium, and other bioactive compounds found in dairy may have beneficial effects on metabolic health and energy balance, thus contributing to weight management. High dietary calcium content found in dairy is essential for regulating energy metabolism. Other Studies have also shown that diets rich in calcium, such as those derived from dairy sources, can help attenuate adipocyte lipid accumulation and weight gain, particularly when consumed alongside energy-dense diets [41, 42]. High-calcium diets have been found to enhance lipolysis and preserve thermogenesis during caloric restriction, facilitating more effective weight loss efforts [43]. However, conflicting evidence exists regarding the relationship between high-fat dairy consumption and obesity risk. While some studies have suggested a positive association between high-fat dairy intake and obesity, others have found no significant correlation. A study conducted by Mozaffarian and colleagues investigated changes in diet and lifestyle and their impact on long-term weight gain in adults [44]. The study observed that individuals with higher intake of high-fat dairy products experienced greater weight gain over time. These products are often high in saturated fats, which have been linked to an increased risk of obesity and related health conditions when consumed in excess. The relationship between dairy consumption and adult obesity is complex and multifaceted, influenced by various factors such as the type of dairy consumed, overall dietary patterns, lifestyle factors, and individual differences. Further research is needed to better understand the mechanisms underlying this relationship and to develop evidence-based recommendations for dairy consumption in the context of obesity prevention and management. In addition to dairy, the consumption of tea and coffee has also been associated with a lower risk of obesity, according to both epidemiological evidence and experimental studies [45, 46]. The beneficial effects of tea are attributed to its rich content of phenolic compounds, which act as potent antioxidants and scavengers of free radicals [47].These compounds contribute to the overall health-promoting properties of tea, including its potential to support weight management efforts. Moreover, this dietary pattern exhibited a high level of red meat consumption, a factor that has been extensively studied in relation to obesity [48]. Numerous research studies have highlighted the association between red meat intake and obesity [49, 50]. In contrast, some studies also highlighted that red meat consumption is not associated with general obesity [51, 52]. In our current investigation, we observed a negative association between red meat consumption and obesity, this result was obtained before adjusting for the potential confounders. The apparent contradiction in findings may be attributed to various factors, including the dietary context in which red meat is consumed. It is plausible that a higher dietary pattern score, characterized by a combination of food groups rich in vegetables, fruits, and plant-based protein sources, could counterbalance the potential adverse effects of red meat consumption on obesity risk. This underscores the importance of considering dietary patterns holistically rather than focusing solely on individual food items when assessing their impact on health outcomes. In this study, typical traditional pattern observed was characterized by a high intake of legumes, starchy foods, vegetables, other cereals, fruits, and rice. We found a positive relationship between this dietary pattern and both general obesity and abdominal obesity among female participants. This finding aligns with similar observations reported in other studies conducted among adult populations in Africa [29, 53, 54]. The positive association between this traditional dietary pattern and obesity may be attributed, at least in part, to its unhealthy constituents. Notably, traditional dietary patterns in many African cultures are often characterized by a high consumption of starchy foods and cereals. This traditional dietary pattern reflects the customary food intake habits among the Yoruba population in southwest Nigeria, characterized by the consumption of starchy staples, such as root vegetables, tubers, and swallow food served with soups or sauces, vegetables, animal source foods, and various spices and condiments [55]. In Nigeria, "swallow foods" often refer to food balls that are completely swallowed without being chewed. They are cooked starchy components that are formed into balls and then often served with soups or stews to be dipped into and consumed. Thus, the dietary habits observed in this study population closely mirror the real-life dietary behaviors of adult populations in the southwestern region of Nigeria. Similar traditional dietary patterns have also been identified in various studies conducted among African populations [29, 30, 53, 54]. This pattern is characterized mostly by a large consumption of carbohydrates. A high-carb diet may be positively correlated with both overall and abdominal obesity, according to studies done in different populations [23, 56].

Furthermore, vegetables are considered healthy constituents in the diet, and prevent weight gain through their low energy density and high dietary fiber content. However, in this study, vegetables showed a positive association with obesity. The traditional way of most vegetable intake among the Yoruba people in southwest Nigeria is making of soup/stew which is served with starchy staples (root, tubers and swallow), animal source foods and other condiments which could inhibit the negative effect of vegetables on obesity. We also observed a positive association between the typical traditional dietary pattern and abdominal obesity among females only, with no such association found among males. Evidence from previous studies aligns with our findings, suggesting that females are more likely to have abdominal obesity. Studies conducted in the United States [57], China [58], and India [59] have consistently shown higher rates of abdominal obesity among women. Moreover, the majority of participants in the typical traditional dietary pattern were married and had 1–3 children. Numerous studies have reported that abdominal obesity is more common among married women, possibly due to hormonal changes associated with pregnancy and menopause [60, 61]. Estrogen, a hormone that fluctuates throughout a woman's life, plays a crucial role in fat distribution. During reproductive years, estrogen promotes the accumulation of subcutaneous fat, while after menopause, decreasing estrogen levels lead to an increase in visceral fat, particularly in the abdomen [62]. This hormonal shift contributes to the propensity for abdominal obesity among postmenopausal women. Furthermore, lifestyle factors such as sedentary behavior and reduced physical activity levels among women compared to men may also contribute to the higher prevalence of abdominal obesity. Women typically engage in less physical activity and may burn fewer calories than men, leading to a greater risk of weight gain and abdominal adiposity. We also found that significant portion of the participants were physically active, with more than 70% exceeding the WHO recommendation for weekly physical activity levels [20]. This prevalence of physical activity likely contributes to the lower prevalence of general obesity observed in our survey population. Interestingly, participants in the highest quartile of the typical traditional dietary pattern demonstrated higher levels of physical activity compared to those in other patterns. This association suggests that some individuals may engage in increased physical activity to mitigate the effects of excess weight gained from consuming an unhealthy diet. Conversely, it could also be a causal relationship, whereby the high physical activity levels observed among participants are a result of their occupation, which may lead them to consume more calorie-dense foods from the typical traditional pattern.

In summary, our study revealed the associations between specific dietary patterns and obesity risk. Traditional dietary patterns were found to be positively associated with obesity, indicating that individuals following these patterns were at higher risk of being obese. Conversely, diversified dietary pattern showed negative associations with obesity, suggesting a potential protective effect against excessive weight gain. More so, variations in these dietary patterns and their associations with obesity were observed across different population groups. Factors such as age, gender, socioeconomic status influenced dietary patterns and obesity prevalence. We observed higher prevalence among female participants, those between the age 41–65, and those with higher monthly income. Understanding which dietary patterns are associated with higher or lower obesity risk can guide the development of targeted dietary recommendations and interventions to promote healthier eating habits and reduce obesity rates. Finally, this study can also be used in the development of preventive strategies targeting high-risk populations. By identifying dietary patterns associated with obesity early in adulthood, interventions can be implemented to promote healthier dietary behaviors and prevent the onset of obesity-related health complications later in life.

### Strength and limitations

The present study demonstrates several strengths and limitations. On the positive side, it represents a significant contribution as the first study conducted in Nigeria focusing on the relationship between dietary patterns and both abdominal and general obesity among adults. Additionally, it utilized a large sample size from a cross-sectional nutrition survey, providing robust data for examining the association between dietary patterns and health outcomes using prevalence rates in Nigeria. Furthermore, the study design was rigorous, incorporating various data points such as demographic information, lifestyle factors, dietary habits, food frequency questionnaire (FFQ), anthropometric measurements, and physical activity levels, which allowed for comprehensive analyses. However, there are several limitations that should be acknowledged. Firstly, the cross-sectional nature of the study design limits the ability to establish causality between dietary patterns and the risk of obesity. Additionally, the use of a qualitative FFQ restricted the calculation of nutrient intake, although it provided insights into dietary patterns and helped minimize recall bias through a 7-day recall period.

## Conclusion

The prevalence of obesity among Nigerian adults remains a significant concern. The findings from this study revealed that the diversified traditional pattern was negatively associated with general obesity in both males and females, while the typical traditional pattern was positively associated with general obesity in both genders and abdominal obesity in females only. Based on these results, our study recommends reducing the consumption of high-carbohydrate diets among adults, particularly those above 40 years of age. Additionally, we emphasize the need for further prospective studies to elucidate the causal associations between dietary patterns and the risk of obesity. The insights gleaned from this study have the potential to inform the development of targeted nutritional interventions aimed at preventing obesity in Nigeria. By understanding the dietary patterns associated with obesity risk, public health initiatives can be tailored to promote healthier eating habits and mitigate the burden of obesity in the Nigerian population.

## Data Availability

No datasets were generated or analysed during the current study.

## References

[1] WHO (2000). Obesity: preventing and managing the global epidemic. World Health Organization technical report series..

[2] Ashkan A (2017). Obesity Collaborators. Health effects of overweight and obesity in 195 countries over 25 years. N Engl J Med..

[3] WHO. Commission on ending childhood obesity: Facts and figures on childhood. Obesity. https://www.who.int/end-childhood-obesity/en/. Accessed 15 Jan 2024

[4] WHO (2005). Obesity: preventing and managing the global epidemic. Report of a WHO consultation. World Health Organ Tech Rep Ser.

[5] Young F, Critchley JA, Johnstone LK, Unwin NC (2019). A review of co-morbidity between infectious and chronic disease in Sub Saharan Africa: TB and Diabetes Mellitus, HIV and Metabolic Syndrome, and the impact of globalization. Glob Health.

[6] WHO. World report on disability 2011. Disabil Soc. 2011; 26(5):655–8.

[7] Troesch B (2015). Increased intake of foods with high nutrient density can help to break the intergenerational cycle of malnutrition and obesity. Nutrients.

[8] Popkin BM (2001). The nutrition transition and obesity in the developing world. J Nutr.

[9] Musaiger AO (2012). Prevalence and risk factors associated with nutrition-related noncommunicable diseases in the Eastern Mediterranean region. Int J Gen Med.

[10] WHO. Obesity and Overweight, Factsheet.2015. https://www.who.int/topics/obesity/en/. Accessed 16 Jan 2024

[11] Abubakari AR (2009). Prevalence and Time Trends in Obesity among Adult West African Populations: A Meta-Analysis. Obes Rev.

[12] Thompson-McCormick JJ, Thomas JJ, Bamivualiku A, Nishakhan A, Becker AE (2010). Breakfast skipping as a risk correlate of overweight and obesity in school going ethnic Fijian adolescent girls. Asia Pac J Clin Nutr.

[13] Olumakaiye MF (2010). Snacking as a Contributor to Overweight among Nigerian undergraduate students. Nigerian J Nutr Sci.

[14] Edwards JS (2015). Changes in food neophobia and dietary habits of international students. J Hum Nutr Diet.

[15] Shu L, Zheng PF, Zhang XY, Gao W (2015). Association between Dietary Patterns and the Indicators of Obesity among Chinese: A Cross-Sectional Study. Nutrients.

[16] Adeoye IA, Okekunle AP (2022). Dietary patterns and associated factors among pregnant women in Ibadan, Nigeria: Evidence from Ibadan pregnancy cohort study. PLoS ONE.

[17] Ambrosini GL, Oddy WH, Robinson M, O’Sullivan TA, Hands BP, de Klerk NH (2009). Adolescent dietary patterns are associated with lifestyle and family psychosocial factors. Publ Health Nutr.

[18] Omage K, Omuemu VO (2018). Assessment of dietary pattern and nutritional status of Undergraduate students in a private university in southern Nigeria. Food Sci.

[19] World Health Organization. Waist circumference and waist-hip ratio: report of a WHO expert consultation, Geneva, 8–11 December 2008. https://iris.who.int/bitstream/handle/10665/44583/?sequence=1. Accessed 16 Jan 2024.

[20] WHO. GPAQ: global physical activity questionnaire (version 2.0). Department of Chronic Diseases and Health Promotion. 2010. http://www.who.int/chp/steps/resources/GPAQ_Analysis_Guide.pdf. Accessed 15 July 2023

[21] Mengesha MM, Roba HS, Ayele BH, Beyene AS (2019). Level of physical activity among urban adults and the socio-demographic correlates: A population-based cross-sectional study using the global physical activity questionnaire. BMC Public Health.

[22] Kim J, Ji JH (2012). A rice-based traditional dietary pattern is associated with obesity in Korean adults. J Acad Nutr Diet.

[23] Lin H, Boi T (2023). Dietary patterns of Hispanic elders are associated with acculturation and obesity. J Nutr.

[24] Yuan Y-Q, Li F, Meng P, You J, Wu M, Li S-G (2016). Gender Difference on the Association between Dietary Patterns and Obesity in Chinese Middle-Aged and Elderly Populations. Nutrients.

[25] Zalesin KC (2008). Impact of obesity on cardiovascular disease. Endocrinol Metab Clin N Am.

[26] Thorpe MG (2016). A comparison of the dietary patterns derived by principal component analysis and cluster analysis in older Australians. Int J Behav Nutr Phys Act.

[27] Adebayo R, Balogun M, Adedoyin R, Obashoro-John Y, Bisiriyu L, Abiodun O (2014). Prevalence and pattern of overweight and obesity in three rural communities in southwest Nigeria. Diabetes Metab Syndr Obes.

[28] Chukwuonye II (2015). Body Mass Index, Prevalence and Predictors of Obesity in Urban and Rural Communities in Abia State South Eastern Nigeria. J Diabetes Metab.

[29] Gubbels JS (2013). Physical Activity, Sedentary Behavior, and Dietary Patterns among Children. Current Nutrition.

[30] Sodjinou R (2007). Dietary patterns of urban adults in Benin: relationship with overall diet quality and socio-demographic characteristics. Eur J Clin Nutr.

[31] Oguntona CRB (2002). Food and nutrient intakes by pregnant Nigerian adolescents during the third trimester. Nutrition.

[32] Ukegbu P (2020). Association of Dietary Patterns and Overweight among University Students Southeast. Nigeria Nigerian Journal of Nutritional Sciences.

[33] Chijoke A (2010). Mortality patterns among type 2 diabetes mellitus patients in Ilorin. Nigeria.

[34] Ukegbu P, Ortutu B, Chinaza U, Ojwang A (2023). Socio-demographic characteristics and dietary pattern of community-dwelling adults in Abia State. Nigeria Ghana Med J.

[35] Akarolo-Anthony SN (2012). Pattern of dietary carbohydrate intake among urbanized adult Nigerians. Inter J of Food Sci and Nutri.

[36] Naja F, Hwalla N, Itani L, Karam S, Mehio Sibai A, Nasreddine L (2015). A Western dietary pattern is associated with overweight and obesity in a national sample of Lebanese adolescents (13–19 years): a cross-sectional study. Br J Nutr.

[37] Romero-Polvo AD (2012). Association between dietary patterns and insulin resistance in Mexican children and adolescents. Ann Nutr Metab..

[38] Koirala GP (2009). Dietary patterns in relation to socio-economic and lifestyle characteristics among Greek adolescents: a multivariate analysis. Public Health Nutr.

[39] Shi Z (2011). Dietary pattern and weight change in a 5-year follow-up among Chinese adults: Results from the Jiangsu Nutrition Study. Br J Nutr.

[40] Lu L, Xun P, Wan Y, He K, Cai W (2016). Long-term association between dairy consumption and risk of childhood obesity: a systematic review and meta-analysis of prospective cohort studies. Eur J Clin Nutr.

[41] Zemel MB, Miller SL (2004). Dietary Calcium and Dairy Modulation of Adiposity and Obesity Risk. Nutr Rev.

[42] Zemel MB, Thompson W, Milstead A, Morris K, Campbell P (2004). Calcium and Dairy Acceleration of Weight and Fat Loss during Energy Restriction in Obese Adults. Obes Res.

[43] Zemel MB (2004). Role of calcium and dairy products in energy partitioning and weight management. Am J Clin Nutr.

[44] Mozaffarian D, Hao T, Rimm EB, Willett WC, Hu FB (2010). Changes in diet and lifestyle and long-term weight gain in women and men. N Engl J Med.

[45] Nagao T (2005). Ingestion of a tea rich in catechins leads to a reduction in body fat and malondialdehyde-modified LDL in men. Am J Clin Nutr.

[46] Dufresne CJ; FER (2010). A review of latest research findings on the health promotion properties of tea. J Nutr Biochem.

[47] Thielecke F (2009). The potential role of green tea catechins in the prevention of the metabolic syndrome—A review. Phytochemistry.

[48] Vergnaud (2010). Meat consumption and prospective weight change in participants of the EPIC-PANACEA study. Am J Clin Nutr..

[49] Schulz M (2002). Food groups as predictors for short-term weight changes in men and women of the EPIC-Potsdam cohort. J Nutr.

[50] Togo P (2001). Food intake patterns and body mass index in observational studies. Int J Obes Relat Metab Disord.

[51] Vergnaud AC (2010). Meat consumption and prospective weight change in participants of the EPIC-PANACEA study. Am J Clin Nutr.

[52] Dabbagh-Moghadam A, Mozaffari-Khosravi H, Nasiri M, Miri A, Rahdar M, Sadeghi O (2017). Association of white and red meat consumption with general and abdominal obesity: a cross-sectional study among a population of Iranian military families in 2016. Eating and Weight Disorders-Studies on Anorexia, Bulimia and Obesity.

[53] Auma C (2019). What Can Dietary Patterns Tell Us about the Nutrition Transition and Environmental Sustainability of Diets in Uganda. Nutrients.

[54] Zeba AN. Dietary patterns and physical inactivity, two contributing factors to the double burden of malnutrition among adults in Burkina Faso, West Africa. J Nutr Sci. 2014;3:17–50. 10.1017/jns.2014.11 PMC4473138 26101618

[55] Akarolo-Anthony SN (2012). Pattern of dietary carbohydrate intake among urbanized adult Nigerians. Inter J of Food Sci and Nutri.

[56] Park SH (2010). Dietary carbohydrate intake is associated with cardiovascular disease risk in Korean: Analysis of the third Korea National Health and Nutrition Examination Survey (KNHANES III). Int J Cardiol.

[57] Tauqeer Z, Gomez G, Stanford FC (2018). Obesity in Women: Insights for the Clinician. J Womens Health.

[58] Sun J-Y, Huang W-J, Hua Y, Qu Q, Cheng C, Liu H-L (2022). Trends in general and abdominal obesity in US adults: Evidence from the National Health and Nutrition Examination Survey (2001–2018). Front Public Health.

[59] Chaudhary M, Sharma P (2023). Abdominal obesity in India: analysis of the National Family Health Survey-5 (2019–2021) data. The Lancet Regional Health - Southeast Asia.

[60] Handayani M, Nadya Putri A, Eva Yani I, Hasniyati R, Sidiq R (2020). Central Obesity Incidence in Adult Women. Int J Med Sci Clin Invent.

[61] Mogre V, Nyaba R, Aleyira S (2014). Lifestyle Risk Factors of General and Abdominal Obesity in Students of the School of Medicine and Health Science of the University of Development Studies, Tamale. Ghana ISRN Obes.

[62] Shi H, Clegg DJ (2009). Sex differences in the regulation of body weight. Physiol Behav.

